# Patient-reported vision impairment in low luminance relates to visual function in age-related macular degeneration: A MACUSTAR study report

**DOI:** 10.1038/s41598-025-14553-4

**Published:** 2025-10-09

**Authors:** Jan Henrik Terheyden, Charlotte Behning, Hannah M. P. Dunbar, Stephen Poor, Nadia Zakaria, Alison M. Binns, Marlene Saßmannshausen, Sergio Leal, Matthias Schmid, Frank G. Holz, David P. Crabb, Ulrich F. O. Luhmann, Robert P. Finger, MACUSTAR consortium, H. Agostini, I. D. Aires, L. Altay, R. Atia, F. Bandello, P. G. Basile, J. Batuca, C. Behning, M. Belmouhand, M. Berger, A. Binns, C. J. F. Boon, M. Böttger, J. E. Brazier, C. Carapezzi, J. Carlton, A. Carneiro, A. Charil, R. Coimbra, D. Cosette, M. Cozzi, D. P. Crabb, J. Cunha-Vaz, C. Dahlke, H. Dunbar, S. Esposti, R. P. Finger, E. Fletcher, M. Gutfleisch, F. Hartgers, B. Higgins, J. Hildebrandt, E. Höck, R. Hogg, F. G. Holz, C. B. Hoyng, A. Kilani, J. Krätzschmar, L. Kühlewein, M. Larsen, S. Leal, Y. T. E. Lechanteur, U. F. O. Luhmann, A. Lüning, N. Manivannan, I. Marques, C. Martinho, K. P. Moll, Z. Mulyukov, M. Paques, B. Parodi, M. Parravano, S. Penas, T. Peters, T. Peto, M. Pfau, S. Priglinger, S. Ramamirtham, S. Ribeiro, D. Rowen, G. S. Rubin, J. Sahel, C. Sánchez, O. Sander, M. Saßmannshausen, M. Schmid, S. Schmitz-Valckenberg, J. Siedlecki, R. Silva, E. Souied, G. Staurenghi, J. Tavares, D. J. Taylor, J. H. Terheyden, A. Tufail, P. Valmaggia, M. Varano, A. Wolf, N. Zakaria

**Affiliations:** 1 Department of Ophthalmology, University Hospital Bonn, Bonn, Germany; 2 Institute for Medical Biometry, Informatics and Epidemiology, University Hospital Bonn, Bonn, Germany; 3 UCL Institute of Ophthalmology, University College London, London, UK; 4 Novartis Pharma AG, Basel, Switzerland; 5 Division of Optometry and Visual Sciences, School of Health Sciences, City, University of London, London, UK; 6 Bayer AG, Berlin, Germany; 7 Roche Pharmaceutical Research and Early Development, Translational Medicine Ophthalmology, Roche Innovation Center Basel, Basel, Switzerland; 8 Department of Ophthalmology, University Hospital Mannheim, University of Heidelberg, Theodor-Kutzer-Ufer 1-3, 68167 Mannheim, Germany; 9 Centre Hospitalier Intercommunal de Creteil (HIC), University Eye Clinic, CentreHospitalier Creteil, Paris, France; 10 STZ Biomed & STZ Eyetrial at the Center of Ophthalmology, University Hospital Tuebingen, Tuebingen, Germany; 11 Ludwig-Maximilians-Universitaet Muenchen (LMU), University Eye Hospital Munich, Munich, Germany; 12 G. B.Bietti Eye Foundation-IRCCS, Rome, Italy; 13 Department of Ophthalmology, Universitaetsklinikum Freiburg (UKLFR), University of Freiburg, Freiburg, Germany; 14 Department of Ophthalmology Rigshospitalet-Glostrup, Copenhagen University Glostrup, Copenhagen, Denmark; 15 Department of Ophthalmology Luigi Sacco Hospital, University of Milan, Milan, Italy; 16 Ophthalmology and Vision SciencesThe Queen’s University an Royal Group of Hospitals Trust Belfast, Belfast, Northern Ireland; 17 Department of Ophthalmology, Universityof Ulm, Ulm, Germany; 18 Clinical Trial Unit, Department of Ophthalmology Gloucestershire Hospitals, NHS Foundation TrustCheltenham, Cheltenham, UK; 19 Department of Ophthalmology, University Vita Salute-Scientific Institute of San Raffael, Milan, Italy; 20 Universität zu Köln, Zentrum für Augenheilkunde, Cologne, Germany; 21 Department of Ophthalmology, Leiden University Medical Center, Leiden, The Netherlands; 22 Department of Ophthalmology, St. Franziskus Hospital, Münster, Germany; 23 BAYER AG, Leverkusen, Germany; 24 F. Hoffmann-La Roche Ltd, Basel, Switzerland; 25 European Clinical Research Infrastructure Network (ECRIN), Paris, France; 26 AIBILI Associaton for Innovation and Biomedical Research on Light and Image (AIBILI), Coimbra, Portugal; 27 Department of Ophthalmology Porto Medical School, Centro Hospitalar de Sao Joao EPE (Hospital Sao Joao) Hospital S. Joao Porto, Porto, Portugal; 28 Centre Hospitalier National d’Opthalmologie des Quinze-Vingts, Paris, France; 29 Moorfields Eye Hospital NHSFoundation Trust (MBRC), London, UK; 30 Radboud University Medical Center (RUMC), Radbound University, Nijmegen, The Netherlands; 31 City University London, London, UK; 32 Fondation Voir et Etendre, Paris, France; 33 University of Sheffield, Sheffield, UK; 34 University College London (UCL), London, UK; 35 Carl Zeiss Meditec, AG, Jena, Germany; 36 Medical Faculty, Institute for Medical Biometry, Informatics and Epidemiology, University of Bonn, Bonn, Germany; 37 Radboud University Medical Center (RUMC), Radbound University, Nijmegen, The Netherlands; 38 GRADE Reading Center, University Hospital Bonn, Bonn, Germany

**Keywords:** Vision-related quality of life, Age-related macular degeneration, Visual function, Validation, Retina, Clinical trial design

## Abstract

Early stages of age-related macular degeneration (AMD) can lead to a number of visual function deficits, but the patient relevance of these deficits is largely unknown. We therefore investigated how bilateral visual function domains affected by age-related macular degeneration (AMD) are associated with patient-reports. Using data from the cross-sectional part of the MACUSTAR study with 245 individuals with AMD (34 early AMD, 168 intermediate (i) AMD, 43 late AMD), the Vision Impairment in Low Luminance (VILL) questionnaire (subscales: reading, VILL_R; mobility, VILL_M; emotional well-being, VILL_E) and visual function assessments from both eyes (best-corrected and low-luminance visual acuity, BCVA, LLVA; Moorfields acuity, MA; contrast sensitivity, CS) were included. Associations between VILL and visual function data (better and worse eyes defined based on BCVA) were investigated using age- and sex-adjusted regression models. In the overall sample, VILL_R and VILL_M were associated with all functional tests across eyes (p ≤ 0.0389), while VILL_E was associated with MA and CS (p ≤ 0.0302). Regression estimates for BCVA, LLVA, MA and CS in the better-seeing eyes were -2.70, -1.84, -1.83 and 1.08 (VILL_R); -2.71, -1.87, -1.90 and 1.88 (VILL_M), and -0.25, -0.22, -2.15 and 1.57 (VILL_E). In iAMD, CS and MA in the worse-seeing eye were associated with two VILL subscales, respectively (VILL_R and VILL_M; VILL_M and VILL_E, respectively; p ≤ 0.0395), while BCVA and LLVA in the worse-seeing eye were both associated with one VILL subscale (VILL_M; p ≤ 0.0317). CS in the better eye was associated with VILL_M (p = 0.0454). Thus, patient-reported outcomes are associated with visual function assessments in both eyes in people with AMD. Contrast vision seems particularly patient-relevant in iAMD. Our results further support the construct validity of the VILL questionnaire.

## Introduction

Age-related macular degeneration (AMD) is a common global cause of blindness and is associated with vision impairment, falls, loss of independence and depression^1,2^. Although treatments are currently available only for late AMD – i.e. the disease stage when such outcomes occur^3,4^ – reductions in visual function and vision-related quality of life already appear in earlier AMD stages^5,6^. Visual domains affected in early and intermediate AMD include night vision, contrast vision, and dark adaptation, which are rarely assessed in routine patient care and large-scale clinical trials. Also, multiple visual function assessments are time- and cost-intensive and difficult to implement in trials. Patient-reported outcome measures (PROMs), on the other hand, are easy to obtain on site or remotely, standardized, and measure concepts relevant to patients, which is increasingly requested by regulatory authorities^7^.

The Vision Impairment in Low Luminance (VILL) questionnaire has recently been introduced and validated to assess vision-related quality of life in AMD^6,8,9^. It overcomes disadvantages of other previously used instruments. The VILL was developed in accordance with the requirements of the United States Food and Drug Administration (FDA) guideline for PROM development^10^ and has been shown to be internally consistent, test–retest reliable, inter-mode reliable, content valid, and able to differentiate between different AMD stages^6,8,9^.

Several visual function assessments including best-corrected and low-luminance visual acuity, low luminance deficit, contrast sensitivity, Moorfields acuity, and rod intercept time from adaptometry^11–15^ are associated with vision-related quality of life and patient-reported symptoms of AMD. However, most of these data were obtained from visual function assessments of only one eye, which limits the ecological (i. e., ‘real-life’) external validity of these analyses.

Despite increasing recognition of the importance of patient-reported outcomes in AMD, evidence supporting the construct validity of newer, specific instruments like the VILL questionnaire remains limited. In particular, associations between bilateral functional vision measures and patient-reported difficulties under low luminance have not been thoroughly investigated, leaving a critical gap in validating such tools for use in clinical trials. We thus analyzed the association between vision-related quality of life, as assessed by the VILL questionnaire, and visual function in both eyes in the MACUSTAR study, a multi-centre European cohort study on intermediate AMD and neighbouring disease stages.

## Methods

### Participants

We included participants of the cross-sectional part of the MACUSTAR study. The MACUSTAR study is a multi-centre cohort study investigating functional, structural and patient-reported outcomes in intermediate AMD. It is conducted at 20 study sites across seven European countries, from which 18 sites recruited participants for the cross-sectional part referred to in this report^16,17^.

In summary, 586 participants with intermediate AMD and 133 control participants with bilateral early AMD (n = 34), late AMD (n = 43) or no AMD (n = 56) were recruited for the MACUSTAR study from early 2018. The disease categorization of AMD followed the Beckman classification^18^. Comprehensive six- to twelve-monthly assessments including visual function testing, multimodal imaging and administration of PROMs were performed in all participants with intermediate AMD or early AMD within the longitudinal part of MACUSTAR.

Within the cross-sectional part of the MACUSTAR study, 168 participants with bilateral intermediate AMD and all control participants attended an additional retest visit scheduled 2 weeks (± 1 week) after the baseline visit. Acquisition of functional, structural and patient-reported data followed procedure manuals specifically developed for the study and all functional data underwent a formal, pre-specified review procedure on a semi-annual basis to ensure that the multi-centre setting of MACUSTAR does not compromise data quality. Imaging data were graded by a central reading centre (GRADE Reading Center, University Hospital Bonn, Germany)^16,17^.

We included only participants with AMD in our analysis and thus excluded healthy controls (Supplementary Figure). We also excluded participants who only participated in the longitudinal part, where disease stage was allowed to be asymmetric.

The study protocol and additional information on the conduct of the MACUSTAR study have been published previously^16,17^ and can be found on the study website www.MACUSTAR.eu. Ethics committees at all participating study sites approved the conduct of the MACUSTAR study and it followed the tenets of the Declaration of Helsinki. All participants provided written informed consent. The clinicaltrials.gov registration identifier is NCT03349801.

### Vision Impairment in Low Luminance (VILL) questionnaire

The VILL questionnaire is a 33-item PROM assessing vision-related quality of life in individuals with AMD, specifically designed for future use in intermediate AMD clinical trials. Its item pool was developed with extensive patient input (in-depth interviews, focus group discussions, cognitive debriefs in people with AMD) and it focuses on the visual difficulties in low-luminance and low-contrast situations which is frequently reported by people with AMD^8^. An initial 37 item-version of the VILL was reduced to a final set of 33 items based on the MACUSTAR data^6,8^. The VILL includes items from the three independent subscales “reading and accessing information” (17 items, “reading”), “mobility and safety (12 items, “mobility”) and “emotional well-being” (4 items, “emotional”). The response scale of the VILL includes four response options per item and a separate response option ”not applicable”. The VILL is scored through Rasch models which have been established and psychometrically validated in the MACUSTAR cohort previously^6,8^.

The VILL has been shown to be an internally consistent, test–retest reliable, content and construct valid PROM which yields similar scores across modes of administration (interview administration, self-administration via paper and electronic self-administration)^6,9^. Within MACUSTAR, the VILL was administered in a standardized manner, using a PROM administration manual by the clinical site personnel in the respective local language^6,17^.

### Visual function assessments

Visual function assessments implemented in the MACUSTAR study include best-corrected (BCVA) and low-luminance visual acuity (LLVA), Moorfields acuity (MA), Pelli-Robson chart contrast sensitivity (PR-CS), mesopic and scotopic fundus-controlled perimetry (microperimetry; S-MAIA, CenterVue, Padova, Italy) as well as dark adaptometry (AdaptDx, Maculogix, Pennsylvania, USA). The assessments available from both eyes were selected as main read-outs for in this study, i.e. BCVA [logMAR], LLVA [logMAR], MA [logMAR] and PR-CS [logCS], which are all chart-based. In a supplemental analysis, average threshold from mesopic and scotpic microperimetry (mAT and sAT [dB]) as well as rod intercept time from dark adaptometry (RIT [min]) were also considered. However, mAT, sATm, and RIT were only available for one eye, which was not necessarily the better eye (i.e., the eye with higher BCVA). Reading performance was also assessed in the MACUSTAR study but not considered in our analysis due to the performance-based nature of reading speed assessments, contrasting the other visual function metrics assessed.

All functional testing procedures were conducted by specifically trained and tested staff that followed specific testing protocols as reported previously^5,17,19^. Visual function testing was conducted separately on both eyes in the MACUSTAR study. Following the study protocol, both eyes had the same AMD stage. Previous research supports that visual function of both eyes impacts vision-related quality of life^20–23^, which was the rationale for including only the visual function assessments that were available from both eyes in our main analysis. In a supplemental analysis, data from only the study eyes were considered, which were selected based on better visual acuity at screening if both eyes were considered eligible.

### Statistical analysis

To investigate the relationship between the VILL subscale person measures and the above-mentioned visual function parameters of both eyes in all participants with AMD, we fitted linear regression models with VILL person measures as dependent variables and the respective functional parameters as independent variables, controlling for age, sex, and AMD stage. Next, we fitted linear regression models using the same approach in the intermediate AMD subgroup, which is the main target population of the MACUSTAR study. All analyses were conducted using R (version 4.3.0, R Core Team, Vienna, Austria). P-values < 0.05 were considered statistically significant.

## Results

We included 245 participants with AMD in our analysis (early AMD, n = 34, 79% female, mean age 72 ± 6 years; intermediate AMD, n = 168, 65% female, mean age 71 ± 8 years; late AMD, n = 43, 64% female, mean age 72 ± 7 years). Mean logMAR BCVA was 0.15 ± 0.32 for study eyes and 0.20 ± 0.36 for fellow eyes (Table 1).

**Table 1:** PROM and visual function data from all included participants (n = 245).

		**Mean ± SD or n(%)**
Sex	Female	157 (64.1%)
	Male	88 (35.9%)
Age		71.9 ± 7.2
AMD stage	Early	34 (13.9%)
	Intermediate	168 (68.6%)
	Late	43 (17.6%)
VILL-Reading^#^		1.5 ± 2.2
VILL-Mobility^#^		1.5 ± 2.3
VILL-Emotional^#^		1.3 ± 3.7
BCVA, logMAR	Better eye	0.08 ± 0.23
	Worse eye	0.27 ± 0.40
LLVA, logMAR	Better eye	0.30 ± 0.27
	Worse eye	0.45 ± 0.37
MA, logMAR	Better eye	0.50 ± 0.24
	Worse eye	0.62 ± 0.31
PR-CS, logCS	Better eye	1.51 ± 0.25
	Worse eye	1.43 ± 0.34

^#^ Person measures.

BCVA = best-corrected visual acuity; LLVA, low-luminance visual acuity; MA, Moorfields acuity; PR-CS, Pelli-Robson contrast sensitivity; VILL, Vision Impairment in Low Luminance questionnaire.

### Vision-related quality of life and visual function across AMD stages

**Fig. 1:**
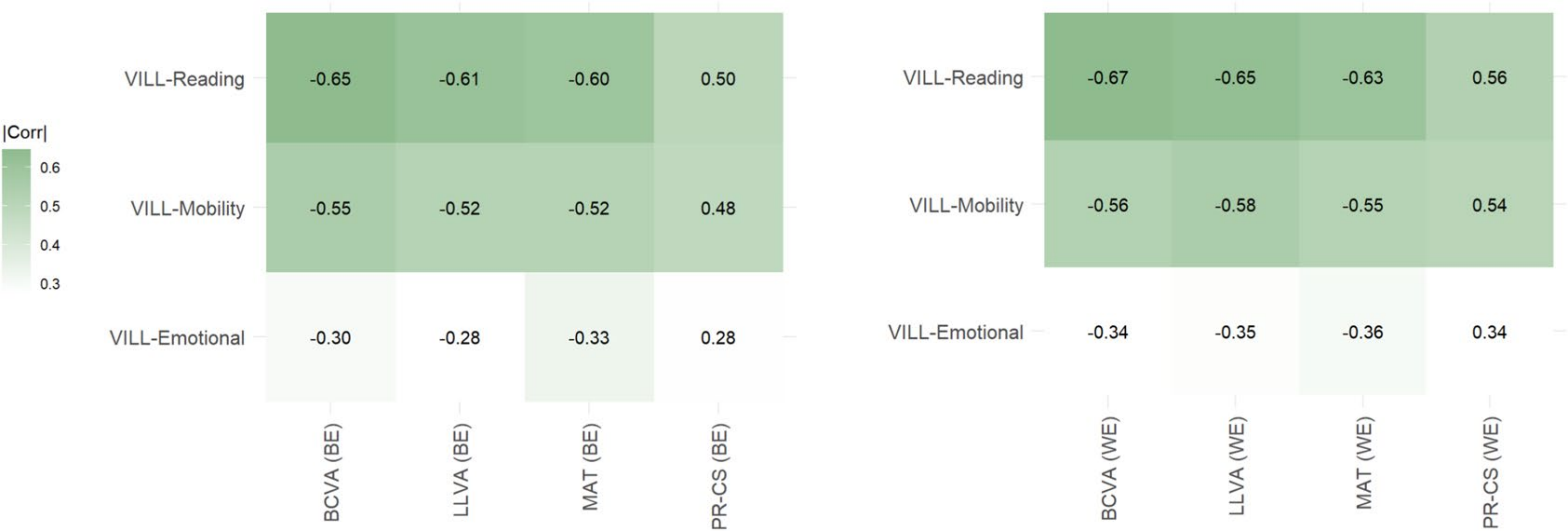
Correlation heatmap of VILL subscale scores (displayed in rows) and visual function testing results from both eyes (columns) across AMD stages (n = 245). BCVA, best-corrected visual acuity; BE, better eye; LLVA, low-luminance visual acuity; MAT, Moorfields acuity test; PR-CS, Pelli-Robson contrast sensitivity; WE, worse eye.

Absolute Pearson correlation coefficients between VILL subscale scores and chart-based visual function parameters from both eyes in all 245 participants with AMD ranged between 0.50 and 0.67 for the VILL reading subscale, between 0.48 and 0.58 for the mobility subscale and between 0.28 and 0.36 for the emotional subscale (Fig. 1). When introducing these variables into a regression model adjusted for age, sex and AMD stage, all visual function parameters were significantly associated with the VILL reading, mobility, or emotional subscale scores (Table 2). Specifically, BCVA, LLVA, MA and PR-CS were associated with VILL-Reading and VILL-Mobility in both the better and the worse eye. VILL-Emotional was associated with MA and PR-CS in the worse-seeing eye.

**Table 2:** Linear regression analysis of functional assessments (independent variables) in all participants with AMD (n = 245) against VILL subscales (dependent variables).

	**VILL-Reading**		**VILL-Mobility**		**VILL-Emotional**	
	β [95% CI]	p-value	β [95% CI]	p-value	β [95% CI]	p-value
Better eye						
BCVA, logMAR	**−2.70 [−4.09; −1.31]**	**0.0002**	**−2.71 [−4.32; −1.11]**	**0.001**	−0.25 [−3.62; 2.57]	0.7401
LLVA, logMAR	**−1.84 [−2.99; −0.69]**	**0.0018**	**−1.87 [−3.19; −0.55]**	**0.0057**	−0.22 [−2.75; 2.31]	0.8645
MA, logMAR	**−1.83 [−3.16; −0.51]**	**0.0068**	**−1.90 [−3.43; −0.38]**	**0.0146**	**−2.15 [−5.05; 0.74]**	0.1443
PR-CS, logCS	**1.08 [0.06; 2.10]**	**0.0389**	**1.88 [0.72; 3.03]**	**0.0016**	**1.57 [−0.65; 3.79]**	0.1645
Worse eye						
BCVA, logMAR	**−1.68 [−2.66; −0.69]**	**0.0009**	**−1.83 [−2.96; −0.70]**	**0.0016**	−1.10 [−3.27; 1.07]	0.3194
LLVA, logMAR	**−1.58 [−2.55; −0.62]**	**0.0014**	**−2.35 [−3.44; −1.25]**	** < 0.0001**	−1.65 [−3.77; 0.48]	0.1284
MA, logMAR	**−1.54 [−2.64; −0.44]**	**0.0061**	**−2.14 [−3.39; −0.89]**	**0.0009**	**−2.66 [−5.05; 0.28]**	**0.0289**
PR-CS, logCS	**0.97 [0.13; 1.80]**	**0.0238**	**1.84 [0.90; 2.78]**	**0.0002**	**2.01 [0.19; 3.82]**	**0.0302**

BCVA = best-corrected visual acuity; LLVA, low-luminance visual acuity; MA, Moorfields acuity; PR-CS, Pelli-Robson contrast sensitivity; VILL, Vision Impairment in Low Luminance questionnaire. Rows marked bold were statistically significant.

### Vision-related quality of life and visual function in intermediate AMD

In the subgroup of 168 participants with intermediate AMD, BCVA, LLVA, MAT and PR-CS were associated with VILL mobility subscale scores in the worse-seeing eye when controlling for age and sex (Table 3). PR-CS was also significantly associated with patient-reports in the better eye (i.e., the VILL mobility subscale) and with the VILL reading subscale, while MAT was associated with the VILL emotional well-being subscale (Table 3). Notably, effect sizes were generally larger in models that included visual function data from the worse eye compared to the better eye.

**Table 3:** Linear regression analysis of summed functional assessments (independent variables) in all participants intermediate AMD (n = 168) against VILL subscales (independent variables), controlled for age and sex.

	**VILL-Reading**		**VILL-Mobility**		**VILL-Emotional**	
	β [95% CI]	p-value	β [95% CI]	p-value	β [95% CI]	p-value
Better eye						
BCVA, logMAR	−0.27 [−2.87; 2.32]	0.8350	−1.68 [−4.71; 1.35]	0.2744	−5.04 [−10.33; 0.25]	0.0616
LLVA, logMAR	0.55 [−1.16; 2.26]	0.5285	0.20 [−1.80; 2.20]	0.8433	−1.74 [−5.26; 1.77]	0.3292
MA, logMAR	0.27 [−1.61; 2.15]	0.7800	−0.28 [−2.48; −1.92]	0.8027	−3.35 [−7.19; 0.49]	0.0867
PR-CS, logCS	1.16 [−0.34; 2.67]	0.1277	**1.79 [0.04; 3.54]**	**0.0454**	1.41 [−1.70; 4.52]	0.3718
Worse eye						
BCVA, logMAR	−1.35 [−2.71; 0.02]	0.0531	**−1.82 [−3.41; −0.23]**	**0.0252**	−2.40 [−5.22; 0.42]	0.0943
LLVA, logMAR	−1.23 [−2.50; 0.03]	0.0565	**−1.62 [−3.10; −0.14]**	**0.0317**	−2.13 [−4.75; 0.49]	0.1103
MA, logMAR	−1.33 [−2.71; 0.04]	0.0576	**−1.81 [−3.41; −0.20]**	**0.0274**	**−3.36 [−6.17; −0.54]**	**0.0198**
PR-CS, logCS	**1.37 [0.07; 2.67]**	**0.0395**	**2.17 [0.66; 3.67]**	**0.0050**	1.98 [−0.72; 4.67]	0.1495

BCVA = best-corrected visual acuity; LLVA, low-luminance visual acuity; MA, Moorfields acuity; PR-CS, Pelli-Robson contrast sensitivity; VILL, Vision Impairment in Low Luminance questionnaire. Rows marked bold were statistically significant.

In the MACUSTAR study, microperimetry and dark adaptometry were performed only in the study eye. Therefore, we additionally evaluated associations between VILL scores and unilateral visual function in the study eye (Supplementary Table). This analysis revealed significant associations between the VILL reading and emotional well-being subscales and average thresholds on microperimetry.

## Discussion

Our findings show that patient-reported vision impairment in low luminance, as measured by the VILL questionnaire, is generally associated with bilateral visual function deficits across all AMD stages – and specifically in intermediate AMD. This further supports the construct validity of the VILL as a tool for assessing visual functioning and vision-related quality of life in AMD. Additionally, our results underscore contrast sensitivity as a key factor contributing to everyday visual difficulties, particularly in individuals with intermediate AMD.

This is the first study to investigate the association between visual function measures and patient-reported low-luminance vision impairment in individuals with intermediate AMD using the VILL questionnaire. These findings are clinically and regulatorily relevant, as they support the use of the VILL as a potential endpoint for assessing functional vision and vision-related quality of life in this patient population. Other patient-reported instruments assessing general vision-related quality of life, as well as those specifically targeting mesopic and scotopic visual functioning, have previously been compared to visual function measurements^11,24–26^. The Low Luminance Questionnaire (LLQ) was associated with dark adaptation, as reported by Owsley and colleagues^27^. This was consistent with a later study by another group, in which rod intercept time from dark adaptometry was highly associated with LLQ scores, alongside BCVA and contrast sensitivity, which were linked to some of the subdomains^28^. In a more recent paper, LLVA and low luminance deficit – defined as the difference between BCVA and LLVA – were significantly associated with LLQ composite scores, whereas BCVA, rod intercept from dark adaptation, and microperimetry thresholds were not^13^. Wu and coleagues used the 10-item Night Vision Questionnaire (NVQ)^29^ in 100 individuals with bilateral intermediate AMD and found that the PROM scores were not significantly associated with BCVA, LLVA, or retinal sensitivity measured by microperimetry^12^. However, the low luminance deficit was significantly greater in individuals reporting night vision difficulties in their study.

Our analysis adds to these results by showing that chart-based visual function tests were indeed associated with vision-related quality of life across AMD stages, and specifically with visual functioning of the worse eye in intermediate AMD. In contrast to previous studies, we included MAT and PR-CS in addition to BCVA and LLVA. Notably, PR-CS was significantly associated with visual functioning in intermediate AMD (i.e., reading and accessing information, mobility and safety). The association between the VILL’s mobility subscale and PR-CS was significant for both the better and worse eyes. The particular importance of contrast vision is also supported by previous qualitative data^8,30^. In addition to chart-based visual function tests, unilateral mean sensitivity reduction on microperimetry was associated with the reading and emotional well-being subscale scores of the VILL questionnaire in our data, contrasting the findings of Thompson et al. in a smaller cohort with varying disease stages^13^. Dark adaptometry in the study eye, however, did not have significant associations with any of the VILL scores, which could be related to the different testing spot in MACUSTAR compared to other protocols^27^. Interpreting our data in the context of similar PROMs further supports the VILL’s construct validity and suggests that the VILL is a suitable tool for future clinical studies and drug trials in the context of AMD.

The results of our study may serve as a baseline interpretation of the minimally important difference (MID) for the VILL questionnaire, using visual function as an anchor. Regulators commonly accept a three-line change in BCVA as clinically meaningful^7^, which could be translated into patient-relevant differences of 0.41 logits in the VILL-Reading subscale, 0.55 logits in the VILL-Mobility subscale, and 0.72 logits in the VILL-Emotional subscale. Previous data support that visual function in the better-seeing eye – used for this estimation – is a stronger predictor of vision-related quality of life than visual function in the worse-seeing eye^23^. However, this interpretation of our results as MIDs is limited by the absence of longitudinal data and a patient-reported anchor variable.

Our main results are based on the analysis of both eyes, which is known to better reflect vision-related quality of life than monocular vision in the context of visual impairment^20–23^. The particular importance of contrast vision of both eyes for vision-related quality of life in intermediate AMD is strongly supported by our findings, where PR-CS in both better and worse eyes was associated with VILL mobility subscale scores. Similar studies to ours have stratified the ‘better’ and ‘worse’ eyes based on BCVA^11,12,27,28,31^, while the relative value of other AMD-affected function domains (e.g., contrast vision, dark adaptation) to patients remains unknown.

Strengths of our study include the large, multicentric and thoroughly phenotyped sample with the same disease stage in both eyes; a broad battery of visual function tests performed under highly standardized settings by experienced study teams; and the validated VILL questionnaire, which was specifically developed to be used in the context of AMD, using both qualitative and quantitative methods and is scored with Rasch models. Limitations include the lack of binocular visual function testing, which we addressed by separately analysing data from the eye with better and worse BCVA. Some assessments, including microperimetry and dark adaptometry were performed in only one eye, and were thus used for generating supplementary findings only. Lastly, we were unable to directly compare associations between the VILL and visual function assessments with other PROMs, as the VILL is the only ophthalmic PROM included in the MACUSTAR study.

In conclusion, our data show that vision-related quality of life in AMD, as measured by the VILL questionnaire, is associated with a variety of visual function assessments. In intermediate AMD, contrast vision – when considering both eyes – is particularly linked to visual functioning. Our findings support the validity of the VILL questionnaire as a patient-relevant measure for future studies.

## Supplementary Information

Supplementary Information.

## Data Availability

The dataset used for this study cannot be publicly shared to protect the privacy of study participants. The data are available from the MACUSTAR consortium upon reasonable request via e-mail (dataaccess@macustar.eu).

## Declarations

### Disclaimer

The communication reflects the author’s view and neither IMI nor the European Union, EFPIA, or any Associated Partners are responsible for any use that may be made of the information contained therein.
