# Supplementary material for: Patient-reported vision impairment in low luminance relates to visual function in age-related macular degeneration: A MACUSTAR study report
Source: Sci Rep. 2025 Oct 9;15:35223. doi: 10.1038/s41598-025-14553-4 (PMC12511350; doi:10.1038/s41598-025-14553-4)
Supplement: Supplementary file 1 — Supplementary Information. [file 41598_2025_14553_MOESM1_ESM.pdf]

Patient-reported Vision Impairment in Low Luminance Relates to Visual Function in Age-Related Macular Degeneration – a MACUSTAR study report

Jan Henrik Terheyden[1], MD; Charlotte Behning[2], MSc; Hannah M. P. Dunbar[3], PhD;

Stephen Poor[4], PhD; Nadia Zakaria[4], MD, PhD; Alison M. Binns[5], PhD;

Marlene Saßmannshausen[1], MD; Sergio Leal[6], MD; Matthias Schmid[2], PhD;

Frank G. Holz[1], MD; David P. Crabb[5], PhD; Ulrich F.O. Luhmann[7], PhD; Robert P. Finger[1,8], MD, PhD on behalf of the MACUSTAR consortium

SUPPLEMENT

Supplementary Table 1. Linear regression analysis of functional assessments (independent variables) in study eyes of participants with intermediate AMD (n=168) against VILL subscales, controlled for age and sex. Visual function of the non-study eyes was not considered in this analysis due to the availability of data

	**VILL-Reading**		**VILL-Mobility**		**VILL-Emotional**	
	β [95% CI]	p-value	β [95% CI]	p-value	β [95% CI]	p-value
BCVA, logMAR	-0.76 [-3.19; 1.68]	0.5394	-1.36 [-4.20; 1.49]	0.3477	-5.29 [-10.24; -0.33]	**0.0366**
LLVA, logMAR	-0.14 [-1.82; 1.54]	0.8686	0.06 [-1.91; 2.02]	0.9545	-2.42 [-5.85; 1.02]	0.1673
MA, logMAR	0.02 [-1.81; 1.84]	0.9856	-0.42 [-2.56; 1.71]	0.6961	-4.09 [-7.79; -0.38]	**0.0309**
PR-CS, logCS	1.17 [-0.28; 2.63]	0.1135	1.91 [0.21; 3.60]	**0.0274**	1.85 [-1.16; 4.87]	0.2257
Mesopic AT [dB]	0.03 [-0.04; 0.09]	0.3745	0.06 [-0.02; 0.13]	0.1527	0.21 [0.08; 0.34]	**0.0014**
Scotopic AT [dB]	0.06 [0.00; 0.13]	**0.0489**	0.07 [-0.00; 0.15]	0.0522	0.18 [0.06; 0.31]	**0.0051**
Rod Intercept Time [min]	0.02 [-0.04; 0.08]	0.4691	-0.03 [-0.09; 0.04]	0.4286	-0.07 [-0.19; 0.04]	0.2246

AT, average threshold; BCVA= best-corrected visual acuity; LLVA, low-luminance visual acuity; MA, Moorfields acuity; PR-CS, Pelli-Robson contrast sensitivity; VILL, Vision Impairment in Low Luminance questionnaire. Rows marked bold were statistically significant.

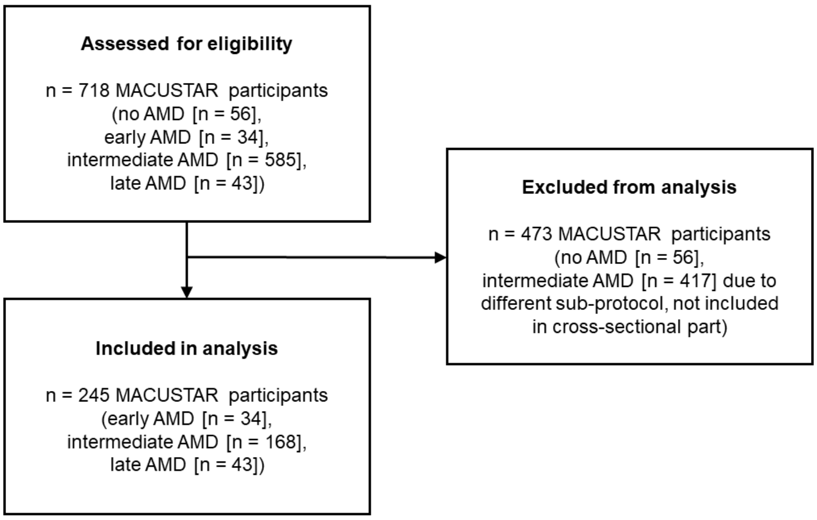

Supplementary Figure 1. Study flow chart

Supplementary Table 2. List of MACUSTAR study sites

**Country**	**Study Site**
Denmark	Rigshospitalet – Glostrup, Department of Ophthalmology Copenhagen University
France	Centre Hospitalier Creteil, University Eye Clinic<br>Centre National d'Ophthalmologie des Quinze-Vingts, Centre d'Investigation Clinique
Germany	University Hospital Tuebingen (UKT), STZ Biomed & STZ Eyetrial at the Center for Ophthalmology<br>University Eye Hospital Munich<br>University of Bonn, Department of Ophthalmology<br>University of Freiburg, Department of Ophthalmology<br>University of Cologne, Department of Ophthalmology<br>University of Ulm, Department of Ophthalmology<br>Department of Ophthalmology, St. Franziskus-Hospital
Italy	G. B. Bietti Foundation – IRCCS<br>Luigi Sacco Hospital, University of Milan, Department of Ophthalmology<br>University Vita Salute – Scientific Institute of San Raffael, Department of Ophthalmology
Netherlands	Department of Ophthalmology, Radboud University Medical Centre<br>Department of Ophthalmology, Leiden University Medical Center
Portugal	AIBILI – Association for Innov. and Biom. Research on Light and Image<br>Oporto Medical School – Hospital S. João, Department of Ophthalmology
United Kingdom	NIHR Moorfields Clinical Research Facility, Moorfields Eye Hospital, NHS Foundation Trust<br>The Queen's University and Royal Group of Hospitals Trust, Ophthalmology and Vision Science<br>Gloucestershire Hospitals NHS Foundation Trust, Clinical Trials Unit, Department of Ophthalmology
